# Alleviation of photoaging‐associated MMP upregulation, prostanoid biosynthesis, and cell cycle arrest with titanium dioxide, zinc oxide, and inorganic‐only (ZnO + TiO_2_ ) sunscreens

**DOI:** 10.1111/php.70043

**Published:** 2025-10-22

**Authors:** Neil Dominic T. Pangilinan, Mohammad Shalbaf, Aline Souza, Bhaven Chavan, Mark A. Birch‐Machin

**Affiliations:** ^1^ Dermatological Sciences, Translational and Clinical Research Institute Newcastle University Newcastle upon Tyne, Tyne and Wear UK; ^2^ Croda Europe Ltd Snaith UK

**Keywords:** photoaging, photoprotection, titanium dioxide, ultraviolet rays, zinc oxide

## Abstract

Exposure to UVR is well understood to accelerate symptoms of photoaging such as wrinkling and loss of skin elasticity. Sunscreen formulations containing titanium dioxide (TiO_2_) and zinc oxide (ZnO) UV filters can therefore be used as an effective photoprotective measure to prevent the induction of signaling pathways in skin that contribute to photoaging. The aim of this study is to provide a broad investigation on the photoprotective impact of TiO_2_, ZnO, and inorganic‐only (ZnO + TiO_2_) sunscreen formulations in human dermal fibroblasts at a gene and protein level. The study focused on genes involved in UV‐only and complete solar light‐induced MMP production, prostanoid biosynthesis for inflammation, and cell cycle arrest, as previously identified through RNA‐seq analysis. Three inorganic formulations were prepared at commercially applicable active levels and varying particle sizes: (1) F(TiO_2_^179nm^), (2) F(ZnO^57nm^), and (3) an inorganic‐only (ZnO + TiO_2_) formulation F(ZnO^57nm^/TiO_2_^47nm^). The three formulations significantly alleviated the irradiation‐induced expression of MMP1, MMP3, PTGS1, PTGES, MDM2, CDKN1A, and CCNE2, with the latter most alleviated by up to 77% (*p* ≤ 0.05). The inorganic‐only (ZnO + TiO_2_) formulation, containing both inorganic UV filters, exhibited the greatest mean or maximum alleviation in 75% of the genes investigated. Protein analyses of MMP1, PTGES, and p21, by immunocytochemistry and Western blot, also showed positive translation of alleviation at a protein level. The study provides further academic and commercial insights on the photoprotective impact of inorganic particles in sunscreens, based on relevant signaling pathways, genes, and proteins that are induced by UV to accelerate photoaging.

## Graphical abstract

Inorganic sunscreens containing the UV filters titanium dioxide (TiO_2_) and/or zinc oxide (ZnO) alleviated various biomarkers of photoaging in human dermal fibroblasts, at a gene and protein level. The study provides novel insights that observe the impact of inorganic photoprotection through the molecular biology of skin.

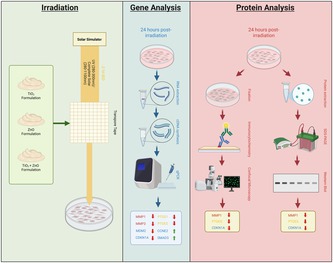

**Abbreviations**
COX2: cyclooxygenase 2
EC: European Commission
ECM: extracellular matrix
MDM2: mouse double minute 2
O/W: oil‐in‐water
PGH2: prostaglandin H2
ROS: reactive oxygen species
SED: standard erythema dose
TiO_2_
: titanium dioxide
US‐FDA: United States‐Food and Drug Administration
UVA: ultraviolet A
UVB: ultraviolet B
UVR: ultraviolet rays
W/O: water‐in‐oil
XRDC: X‐ray diffraction crystallography
ZnO: zinc oxide

## INTRODUCTION

Photoaging of skin, characterized by wrinkling, loss of elasticity, and rough skin texture, is primarily induced by exposure to ultraviolet rays (UVR) emitted from the sun. Although the composition of solar light only includes 5% UVR, ultraviolet A (UVA, 315–400 nm) and ultraviolet B (UVB, 280–315 nm) wavelengths can trigger various acute and chronic effects on the skin, ranging from erythema (sunburn) and pigmentation changes to skin carcinoma and photoaging. It is understood that 80% of extrinsic aging is caused by UVA and UVB^1^, ^2^; therefore, UV wavelengths are key targets for commercial sunscreens which utilize inorganic UV filters that filter harmful UVR radiation.

UV‐induced photoaging can occur through direct or indirect damage to DNA, in both the epidermis and the dermis. However, variance in wavelength and energy stipulates that most UVB is absorbed by the epidermis, while UVA becomes the primary contributor to the dermal stress response. It is generally regarded that characterization of photoaging refers to the influence of UVA on the structure and stability of skin, which implicates the dermis to a greater extent.^3^ Dermal fibroblasts, which comprise the bulk of cell populations within the dermis, are responsible for the production of extracellular matrix (ECM) proteins such as collagens and elastin, which uphold structure and elasticity, respectively. In UV‐damaged human skin, ECM proteins are at lower levels due to the upregulation of ECM‐degrading matrix metalloproteinases such as MMP1 and MMP3, as part of the UV stress response.^4^, ^5^, ^6^, ^7^ MMP1 is one of multiple collagenases that degrade collagen fibers, which provide most of the skin's structural support. Degradation of collagen can be correlated with wrinkling and sagging. Alternatively, degradation of elastin can cause abnormalities in its protein structure and increase the risk of solar elastosis, which causes thickening and yellowing of the skin.^8^, ^9^

The key mechanism for UV damage in the dermis is indirect through overproduction of reactive oxygen species (ROS).^10^ ROS are widely understood to be responsible for activating various pathways in dermal fibroblasts such as AP‐1 and NF‐κB, which accelerate the onset of various photoaging symptoms such as wrinkling through senescence and MMP‐mediated degradation of ECM proteins.^11^, ^12^ There are two theories of how photoaging occurs based on oxidative stress caused by UVA: the Free Radical Theory of Aging and the Vicious Cycle of Damage Theory. The former asserts that aging is synonymous with an accumulation of ROS over time, whereas the latter asserts a positive feedback loop of damage between ROS and cellular components; in other words, ROS‐mediated damage causes production of more ROS.^13^, ^14^

Due to concerns with photoaging, UV, and general sun exposure, sunscreen formulations containing UV‐filtering active ingredients have become a major global commercial business. Physical sunscreens, also known as mineral sunscreens, are one of two major categories (alongside chemical sunscreens) that provide photoprotection. These sunscreens employ inorganic filters such as titanium dioxide (TiO_2_) and zinc oxide (ZnO) to filter UVA and UVB wavelengths by creating a physical barrier on top of the skin that reflects and refracts light. Both inorganic actives can provide broad UVA and UVB protection (280–400 nm). However, TiO_2_ has greater efficacy in the UVB range (280–315 nm), whereas ZnO displays peak protection in the UVA range (315–400 nm), up to 370 nm.^15^, ^16^ When combining TiO_2_ and ZnO particles as an inorganic‐only (ZnO + TiO_2_) formulation, it is reported that there is a broader range of photoprotection against both UVB and UVA.^17^ The United States Food and Drug Administration (US‐FDA) and European Commission (EC) have both set the limit of TiO_2_ and ZnO in sunscreen formulations to 25% of the total formulation concentration.^18^

The use of physical sunscreens has become increasingly popular over the last few decades due to the reduced risk of deep skin penetration and lower degradation rates upon exposure to UV, compared with their chemical counterparts.^19^, ^20^ Moreover, physical sunscreens have been reported to cause less irritation, which increases their suitability for children and adults with sensitive skin.^21^ However, the application of TiO_2_ and ZnO leaves a white cast on the skin due to the high refractive indexes of the particles (TiO_2_: 3.6–4.0; ZnO: 2.3–2.4).^22^, ^23^ This invokes a cosmetic challenge that may affect consumers in Western markets who typically do not want to appear whiter.

In newer formulations, TiO_2_ and ZnO can be used in the nanoparticle scale (<100 nm), which tackles the whitening effect previously observed with larger particles by increasing transparency. The interaction of nanoparticles with UV differs from larger particles as the former demonstrates greater photocatalytic ability. In other words, TiO_2_ and ZnO particles tend to absorb UV rather than reflecting and refracting. It has also been reported that smaller particle sizes tend to sacrifice some UVA protection for increased UVB protection due to a shift in absorbance spectra.^15^

Sunscreen formulation is a complex process that not only accounts for the active ingredients but also multiple ingredients that improve the stability and aesthetics of the cream. By estimate, based on formulations taken from various companies, sunscreens typically consist of 20% active ingredients, 55% formulation stabilizers, 23% sensory enhancers, and 2% added extras.^24^ Consumer considerations, therefore, play a major role in the formulation of sunscreens. A formulation scientist is tasked with ensuring a balance between the level of protection and the appeal of the formulation.

The aim of this study is to investigate the photoprotective impact of TiO_2_, ZnO, and inorganic‐only (ZnO + TiO_2_) sunscreen formulations at a gene and protein level, in the context of photoaging. In a previous study, it was observed that TiO_2_ and ZnO, as dispersions, were able to alleviate the UV‐induced upregulation and downregulation of eight photoaging‐related genes associated with ECM remodeling, prostaglandin‐mediated inflammation, and cell cycle regulation by up to 77%.^25^ The photoaging‐related genes were identified as part of a broad high‐throughput RNA‐seq analysis on HDFn 24 h postirradiation with 2.16 standard erythema dose (SED) UV‐only (280–400 nm) and complete solar light (280–1100 nm). Despite providing abundant insights, the use of dispersions would not be applicable in the real world, since inorganic actives are used as formulations in society. Using similar methodology, UV‐only and complete solar light‐induced expression of the same genes would be investigated in the context of sunscreen formulations. Furthermore, several genes would be further examined by Western blotting and immunocytochemistry to identify whether trends in photoprotection are successfully translated to a protein level, qualitatively and quantitatively. The outcomes of this study would benefit academic advancements on the cellular implications from inorganic‐based photoprotection and commercial outlooks on sunscreen formulation from a biological perspective.

## MATERIALS AND METHODS

### Preparation of sunscreen formulations

Glyceryl stearate/sorbitan stearate sunscreen formulations were prepared with one or more of the three following inorganic UV filters: TiO_2_^179nm^, TiO_2_^47nm^, and ZnO^57nm^. Particle size (nm) was defined as a mean acquired from X‐ray diffraction crystallography (XRDC). TiO_2_ particles were precoated with alumina stearate to reduce photoactivity, following European regulations.^26^ ZnO particles were initially uncoated but were dispersed in an oil carrier using polymeric dispersants, thus being in situ coated.^27^ Three oil‐in‐water (O/W) formulations were prepared with varying inclusion levels of inorganic UV filters, which mimicked existing formulations: (1) 7.35% F(TiO_2_^179nm^), (2) 25% F(ZnO^57nm^), and (3) 21%/2.4% F(ZnO^57nm^/TiO_2_^47nm^). The latter was an inorganic‐only (ZnO + TiO_2_) formulation containing both inorganic UV filters.

An aqueous phase and an oil phase were first prepared, containing the ingredients listed in Table 1. When preparing the aqueous phase, deionized water was added in increments and mixed by hand to form a consistent mixture. The aqueous phase was then mixed for 10 min using an electric stirrer at 600 rpm, upon the addition of all the ingredients. The oil phase was initially prepared without the inorganic UV filters and heated to 80–85°C. The inorganic UV filters were added once the oil phase was hot and mixed for 5 min with an electric stirrer at 500 rpm. Both the aqueous phase and oil phase were heated to 80°C prior to mixing the phases. Under an electric stirrer, the oil phase was slowly added to the aqueous phase and left to emulsify at 1000 rpm for 2 min. The resultant mixture was cooled under a mixer at 600 rpm for 20 min, and 2‐phenoxyethanol preservative was added. The mixture with preservative was left to continue mixing at 600 rpm for a further 5 min. The prepared formulations are described in Table 2.

**TABLE 1:** List of ingredients provided by Croda for the preparation of titanium dioxide and zinc oxide formulations.

Aqueous phase		Oil phase	
Ingredient name	Function	Ingredient name	Function
Deionized Water	Carrier	Glyceryl monostearate	Emulsifier/Moisturizer
Glycerin	Moisturizer	Sorbitan stearate	Emulsifier
Magnesium aluminum silicate	Emulsifier/Thickener	Tween 60	Emulsifier/Thickener
Xanthan gum	Emulsifier/Thickener	Stearyl alcohol	Emulsifier/Thickener
		Caprylic/capric triglyceride	Carrier

Oil phase active ingredients	
Ingredient name	Supplied active concentration (%)
TiO_2_^179nm^	34.3
ZnO^57nm^	57.2
TiO_2_^47nm^	49

*Note*: The formulations require an aqueous phase and oil phase, which are prepared independently and then mixed together. The formulation contains active ingredients, moisturizers, emulsifiers, and thickeners. Active ingredients are added to the oil phase independently.

**TABLE 2:** List of prepared inorganic formulations.

Name	Active ingredients	Mean particle size (nm)	Coating	Active concentration in formulation	Emulsion type	Appearance on Transpore tape
F(TiO_2_^179nm^)	TiO_2_	179	Alumina stearate	7.35%	O/W	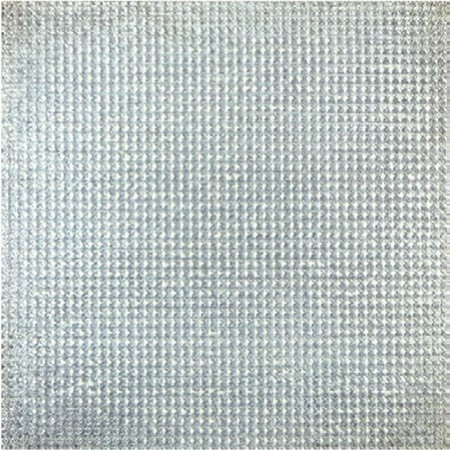
F(ZnO^57nm^)	ZnO	57	Uncoated^a^	25%	O/W	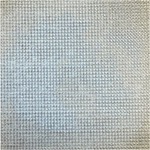
F(ZnO^57nm^/TiO_2_^47nm^)	ZnO/TiO_2_	57/47	Uncoated^a^/Alumina stearate	21.4%/2.4%	O/W	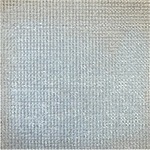

*Note*: All formulations are oil‐in‐water (O/W) emulsions. Active concentrations for each formulation are variable but are based on existing commercial sunscreen formulations. TiO_2_^47nm^ and ZnO^57nm^ are nanoparticles. Titanium dioxide is precoated following European regulations, while ZnO is only coated in situ. Active concentrations require reduction to a maximum of 25% active following European regulations.

^a^ ZnO is coated in situ upon dispersion in an oil carrier using polymeric dispersants.

### Irradiance testing

The irradiance of light passing from the solar simulator and through the creams was measured using a FLAME‐S‐XR1‐ES spectroradiometer with CC‐3‐DA direct‐attach cosine corrector with spectralon diffuser (OceanOptics, Netherlands). The formulations were applied on 5 cm × 5.3 cm Transpore tapes (3 M, USA) with a 2 mg/cm^2^ surface density and spread evenly by finger. Transpore tape was used as it is the established gold standard for irradiation‐based sunscreen testing by Diffey and Robson.^28^ The formulations were left to dry for 30 min to produce an even film of sunscreen on the Transpore tape. The tapes were placed onto PMMA plates which allowed light transmission, suspended over the FLAME spectroradiometer, and placed directly under the solar simulator. Irradiance measurements were taken using the OceanView software, with an integration time of 8 ms and 100 scans. A graphical spectrum with wavelength against absolute irradiance was produced. For each UV filter tested, three recordings were taken, and the data were imported to Microsoft Excel.

### Cell culture

#### Maintenance and seeding of HDFn

HDFn were cultured as 2D monolayers, between passages 5 and 20, in standard DMEM (ThermoFisher Scientific, containing L‐glutamine, phenol red, sodium bicarbonate, and sodium pyruvate) supplemented with 10% fetal bovine serum (FBS) and 1% penicillin/streptomycin. This can be referred to as complete DMEM. The fibroblasts were grown and incubated in T175 flasks at 37°C/5% CO_2_ until passaged or seeded into 35 mm TPP dishes (Corning, USA). HDFn were seeded at a cell density of 35,000 cells per mL. TPP dishes prepared for the next day irradiation were all sealed with parafilm and incubated for 24 h at 37°C/5% CO_2_ in 2 mL complete DMEM prior to irradiation.

#### Irradiation of HDFn with or without photoprotection

The media in the TPP dishes was replaced from complete DMEM to clear DMEM (DMEM with no phenol red, ThermoFisher Scientific, USA, contains high glucose, L‐glutamine, and HEPES) without FBS for irradiation. This ensured that there were no photosensitizers in the media that could interfere with the irradiation. The Newport solar simulator (Newport Oriel model 91292, 1000 W, MKS Instruments, Inc., USA) was used as the light source for irradiation, emitting light from the UVB range to NIR (280–1100 nm). The solar simulator was warmed up for 15 min prior to use and daily variation in output was accounted for using an IL1400A radiometer (Serial 8524 with UVA sensor, serial 867, International Light Technologies, USA). The duration of irradiation was calculated based on the daily variation of output to deliver a 2.16 SED dose, which is equivalent to 20 min of the sun's output at solar noon in midsummer in the Mediterranean at 45° latitude.

The lids of the TPP® dishes were removed during irradiation, as they are known UV absorbers. For photoprotected samples, the lid was replaced with Transpore tape prepared with a film of inorganic sunscreen, as described in Section “Irradiance testing.” The tape was horizontally placed on the rim of the TPP dish to ensure equal distance between the light source above and the HDFn below, ensuring that the tape was suspended in air. Additionally, it was ensured that the Transpore tape remained dry and was not in contact with the irradiation media or HDFn.

HDFns were irradiated with a 2.16 SED dose of complete solar light (280–1100 nm) and UV‐only (280–400 nm) using the solar simulator. UV‐only irradiation was conducted using an IR/VIS blocking filter (UG‐11, Schott, Germany). Nonirradiated controls were covered in aluminum foil and placed under the solar simulator for the same duration as the irradiated samples. Following irradiation, the Transpore tapes were removed from the rim of the dishes and replaced with the lid. Media were replaced to complete DMEM and dishes were incubated for 24 h at 37°C/5% CO_2_.

### Gene Analysis

#### RNA extraction and cDNA synthesis

HDFn were detached from the dishes 24 h after irradiation using trypsin–EDTA (TE). TE was neutralized, and a cell pellet was collected after centrifuging the suspension at 300 × *g* for 5 min. RNA extraction was performed using the RNEasy mini kit (Qiagen, UK), following the manufacturer's instructions. Total RNA concentration was measured using a NanoDrop ND‐100 (Nanodrop Technologies LLC, ThermoFisher Scientific, USA), and RNA purity was assessed using the 260/280 nm ratio provided. The RNA samples were converted to cDNA through reverse transcription using a High‐Capacity cDNA Reverse Transcription Kit (ThermoFisher Scientific, USA) with RNAse inhibitor (ThermoFisher Scientific, USA). Prior to quantitative polymerase chain reaction (qPCR), the samples were diluted to 5 ng/μL and stored at −20°C.

#### Quantitative polymerase chain reaction

qPCR was conducted using TaqMan™ gene expression assays with FAM‐MGB dye label to identify gene expression changes in eight marker genes: MMP1 (Hs00899658_m1), MMP3 (Hs00968305_m1), PTGS1 (Hs00377726_m1), PTGES (Hs00610420_m1), MDM2 (Hs00540450_s1), CDKN1A (Hs00355782_m1), CCNE2 (Hs00180319_m1), and SMAD3 (Hs00969210_m1). Gene expression was identified for unprotected and protected HDFns in both UV‐only and complete solar irradiation conditions. GAPDH (Hs02786624_g1) was used as the housekeeping gene to normalize the observed changes in gene expression. A reaction mixture for qPCR consisted of 1 μL of 20 × TaqMan gene expression assay, 10 μL 2 × TaqMan Fast Advanced Master Mix (4369016, ThermoFisher Scientific, USA), 4 μL of 5 ng/μL DNA in RNase‐free water, and 5 μL of RNase‐free water (20 μL total). qPCR plates were run on Applied Biosystems QuantStudio™3 to amplify cDNA and acquire Ct values for qPCR analysis. Diomni™ Design and Analysis (RUO) 3.0.1 on the ThermoFisher Cloud was used to generate the 96‐well plate plans and qPCR run durations and observe changes in gene expression in real time.

Technical repeats were performed in triplicate for each qPCR run. Three biological repeats were acquired through repetition of the protocol. Mean Ct values were calculated upon acquisition of technical and biological repeats. Fold change was calculated using the ΔΔCt method, which converted Ct values to 2−ΔΔCt, as a measurement of fold change. The cut‐off for Ct values was 35 cycles. The 2−ΔΔCt fold change values were converted to log_2_ values for analysis to identify the directionality in gene expression with respect to the nonirradiated controls.

### Protein Analyses

#### Immunocytochemistry

HDFn were seeded and irradiated using the same approach and conditions as Section “Cell culture.” However, glass bottom dishes (μ‐dish 35 mm, high, Ibidi, Germany) which enabled high resolution confocal images to be taken were used instead of the standard 35 mm TPP dishes. About 24 h postirradiation, samples were washed thrice with PBS and fixed with 4% paraformaldehyde (ThermoFisher Scientific, USA) in a fume hood for 10 min. The fixative was removed by washing with PBS thrice afterwards. Immunostaining was conducted, which involved blocking with 5% v/v goat serum, 0.2% v/v Triton‐X‐100 in PBS, primary antibody incubation with 1% w/v BSA, 0.2% Triton‐X‐100 in PBS, secondary antibody (Invitrogen™ A‐11008) and Alexa Fluor™ 555 Phalloidin (Invitrogen™ A34055) incubation with 1% w/v bovine serum albumin (BSA) and mounting with ProLong™ Diamond Antifade Mountant with DAPI (Invitrogen™ A34055). One of three primary antibodies were applied per run: MMP1 (Invitrogen™ PA5‐27210), PTGES (Invitrogen™ PA5‐60916), or p21(Proteintech™ 10355‐1‐AP).

Imaging was conducted using an AiryScan confocal microscope and Zen Blue V3.8.2. Fluorescence channels for multiple fluorophores were configured to visualize the location of each of the biomarkers within the cell. The fluorescence channels were as follows: DAPI (405 nm excitation and 450 nm emission filter), Alexa Fluor® 488 (488 nm excitation and 525 nm emission filter), and Alexa Fluor® 555 (555 nm excitation and 594 nm emission filter). The channels distinguished the nuclei, biomarker of interest, and F‐actin (cytoplasm), respectively.

Image analyses were conducted using Fiji ImageJ. Depending on the appearance and location of the biomarker, image quantification was applied if possible. For MMP1, quantification was achieved by calculating the number of MMP1‐positive cells, defined as HDFn within the image that produced fluorescence, using the *Cell Counter* tool. For p21, quantification was achieved by measuring mean fluorescence intensity within an established area where fluorescence was observed in every cell. In both quantification instances, nine images were taken and a randomized sample of three images was used to acquire measurements.

#### Protein extraction and separation, Western blotting, and NIR imaging

HDFn in 35 mm standard TPP® dishes were washed once with ice‐cold PBS 24 h postirradiation and lysed with 35 μL RIPA buffer containing protease inhibitor (Pierce® 1× RIPA buffer, ThermoFisher Scientific; Pierce® Protease Inhibitor Mini Tablets, ThermoFisher Scientific). The cells were then scraped using a cell scraper and transferred to 0.5 mL Eppendorfs. The lysates were vortexed and incubated on ice for 20 min, then centrifuged at 4°C for an additional 20 min. The lysate suspensions were transferred to new Eppendorfs and total protein concentration was quantified using the Pierce® Dilution‐Free™ Rapid Gold BCA protein assay (ThermoFisher Scientific), following the supplier's protocol.

Lysates were prepared to a concentration of 1 μg/μL with Laemmli buffer (Bio‐Rad Laboratories, USA) containing 5% β‐mercaptoethanol (ThermoFisher Scientific, USA) at a 1:4 ratio. Proteins in each lysate were separated through SDS‐PAGE, using 4–20% Mini‐PROTEAN TGX gel (Bio‐Rad Laboratories, USA). A Chameleon Duo Pre‐Stained Ladder (LI‐COR, UK) was used as a reference for protein molecular weight. Protein transfer to a nitrocellulose membrane was conducted using the Trans‐Blot® Turbo™ RTA Transfer Kit and was run on a Trans‐Blot® Turbo™ Transfer System (LI‐COR, UK).

Upon transfer, the nitrocellulose membranes were blocked for 1.5 h in 1× TBS‐T buffer containing 5% w/v skimmed milk with gentle agitation, to prevent nonspecific binding. The membranes were subsequently washed thrice with fresh 1× TBS‐T buffer. Primary antibodies were bound to the proteins of interest by incubating the membrane overnight at 4°C with gentle agitation in 1× TBS‐T buffer containing 5% w/v skimmed milk and WB‐compatible primary antibody, diluted between 1:500 and 1:2000 of the original concentration. One of four rabbit polyclonal antibodies was added to the primary antibody solution at this step: GAPDH (Santa Cruz™ sc‐25778), MMP1 (Invitrogen™ PA5‐27210), PTGES (Invitrogen™ PA5‐60916), or p21 (Proteintech™ 10355‐1‐AP).

The membranes were washed with fresh 1× TBS‐T buffer three times on the following day, for 5 min each, to remove unwanted primary antibody residues. Secondary antibody (Goat anti‐rabbit IgG (H + L) cross‐adsorbed secondary antibody, Alexa Fluor™ Plus 488, Invitrogen™ A‐11008) was applied by incubating the membrane for 1 h with gentle agitation in 1× TBS‐T buffer containing 5% w/v skimmed milk and NIR secondary antibody, diluted 1:1000 of the original concentration. Prior to imaging, the membranes were washed three times with fresh 1× TBS‐T buffer for 5 min each to remove unwanted secondary antibody residues that may cause background fluorescence.

Nitrocellulose membranes were imaged using the LI‐COR Odyssey FC imaging system (LI‐COR, UK) and processed on Image Studio Ver 5.2 (LI‐COR, UK). Bands on the nitrocellulose membrane were observed by excitation at 700 and 800 nm, respectively. GAPDH was observed at 36 kDa, MMP1 was observed at 54 kDa, PTGES was observed at 18 kDa, and p21 was observed at 21 kDa. The fluorescent images of the membrane were converted to black and white and transferred to ImageJ for quantitative analysis. Densitometric analysis was conducted on Fiji ImageJ to quantify the bands and normalize to the housekeeping gene, GAPDH.

### Statistical analysis

The statistical significance of quantitative results was assessed using GraphPad Prism 9. One‐way ANOVA was used to compare the means of three or more independent groups in a dataset, followed by Tukey's post hoc test to detect which specific group means were statistically different. The statistical differences of a dataset are graphically represented as: ns = *p* > 0.05, *p ≤ 0.05, **p ≤ 0.01, ***p ≤ 0.001, ****p ≤ 0.0001.

## RESULTS

### TiO_2_ , ZnO, and ZnO + TiO_2_ formulations reduce absolute irradiance transmission in the UVB, UVA, and blue light range

**FIGURE 1:**
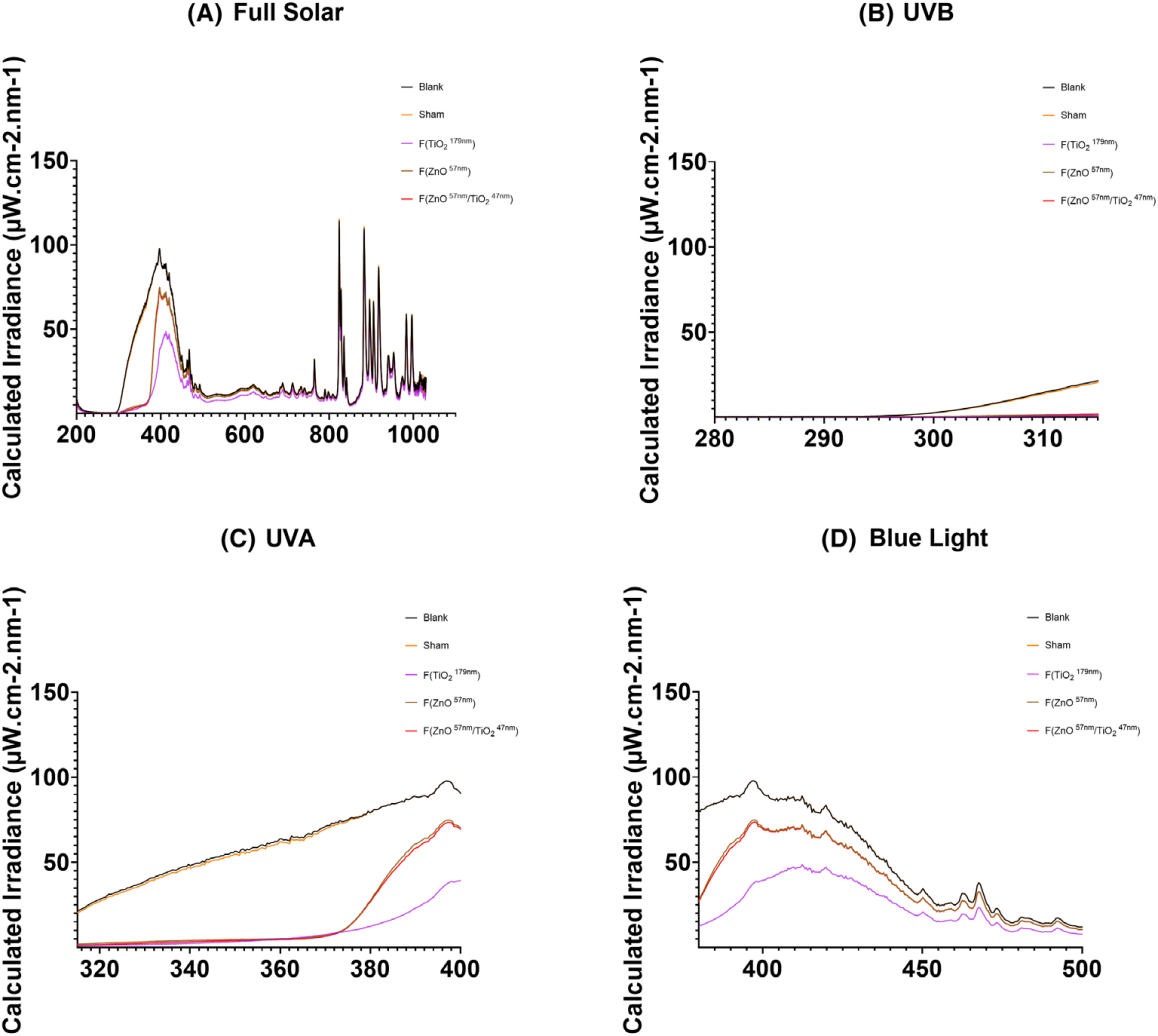
Absolute irradiance from a Newport solar simulator with/without sunscreen formulations. Absolute irradiance was detected using a FLAME spectrometer connected to OceanView software. 2 mg/cm^2^ of each formulation (TiO_2_^47nm^, ZnO^57nm^, TiO_2_^179nm^) were applied onto Transpore tape and placed between the solar simulator and spectrometer. The blank condition represents Transpore tape with no formulation applied and the sham condition represents the use of a moisturizing cream with 0 SPF. (A) Complete solar spectrum 200–1100 nm (B) UVB spectrum 280–315 nm (C) UVA spectrum 315–400 nm (D) Blue light spectrum ~380–500 nm.

An absolute irradiance spectrum across the entire solar spectrum was generated, which compared a blank Transpore tape and a sham cream (0% inorganic filters) to F(TiO_2_^179nm^), F(ZnO^57nm^), and F(ZnO^57nm^/TiO_2_^47nm^; Figure 1). In the presence of the three formulations, a distinct reduction in absolute irradiance was observed across the UVB, UVA, and blue light ranges (Figure 1B–D). All three formulations exhibited similar absolute irradiance values from 290 to 374 nm, which spans most of the UVB range and two‐thirds of the UVA range. From 374 nm, the absolute irradiance trends diverge between the formulations containing only one type of inorganic filter and the inorganic‐only (ZnO + TiO_2_) formulation, F(ZnO^57nm^/TiO_2_^47nm^).

Across the UVB spectrum (280–315 nm), the absolute irradiance for the three formulations consistently remained <3 μW·cm^−2^·nm^−1^; on the contrary, the blank and sham conditions progressively increase with increasing wavelength to ~20 μW·cm^−2^·nm^−1^ at 315 nm. Despite minimal differences in absolute irradiance between the three formulations within the UVB range, the order of the formulations from highest absolute irradiance to lowest is as follows: F(ZnO^57nm^) > F(ZnO^57nm^/TiO_2_^47nm^) > F(TiO_2_^179nm^).

In the UVA range, the three sunscreens maintain this order and show similar absolute irradiance values across UVAII (315–340 nm) and some of UVAII (340 and 374 nm). The absolute irradiance slightly but consistently increases along this wavelength range to ~10 μW·cm^−2^·nm^−1^ at 374 nm. The difference between the controls and the formulations is at its greatest at 374 nm, whereby the absolute irradiance for the former is approximately sevenfold greater. When wavelength > 374 nm, F(ZnO^57nm^) and F(ZnO^57nm^/TiO_2_^47nm^) begin to express a steeper increase in absolute irradiance compared with the formulation with larger inorganic mean particle size, F(TiO_2_^179nm^). Both nanoparticle‐based and ZnO‐containing formulations maintain similar absolute irradiance values to each other, as before <374 nm, despite a greater increase in absolute irradiance.

Peak absolute irradiance is observed between 390 nm and 395 nm for the blank control, sham control, F(ZnO^57nm^), and F(ZnO^57nm^/TiO_2_^47nm^). As the highest absolute irradiance for the blank control, it asserts the peak output of light from the solar simulator ~97 μW·cm^−2^·nm^−1^. The peak absolute irradiance for F(ZnO^57nm^) and F(ZnO^57nm^/TiO_2_^47nm^) is ~75 μW·cm^−2^·nm^−1^. Meanwhile, F(TiO_2_^179nm^) does not produce a peak until 410 and 415 nm, which is no longer considered to be in the UVA range, but rather the blue light range. The peak absolute irradiance for F(TiO_2_^179nm^) is ~48 μW·cm^−2^·nm^−1^. Likewise, with the UVB range, the same order in absolute irradiance from highest to lowest is observed across the UVA, visible light, and NIR range: Blank > Sham > F(ZnO^57nm^) > F(ZnO^57nm^/TiO_2_^47nm^) > F(TiO_2_^179nm^).

### Analyses of eight photoaging‐associated genes

#### TiO_2_ , ZnO, and ZnO + TiO_2_ formulations can alleviate irradiation‐induced MMP1 and MMP3 upregulation to below nonirradiated levels

**FIGURE 2:**
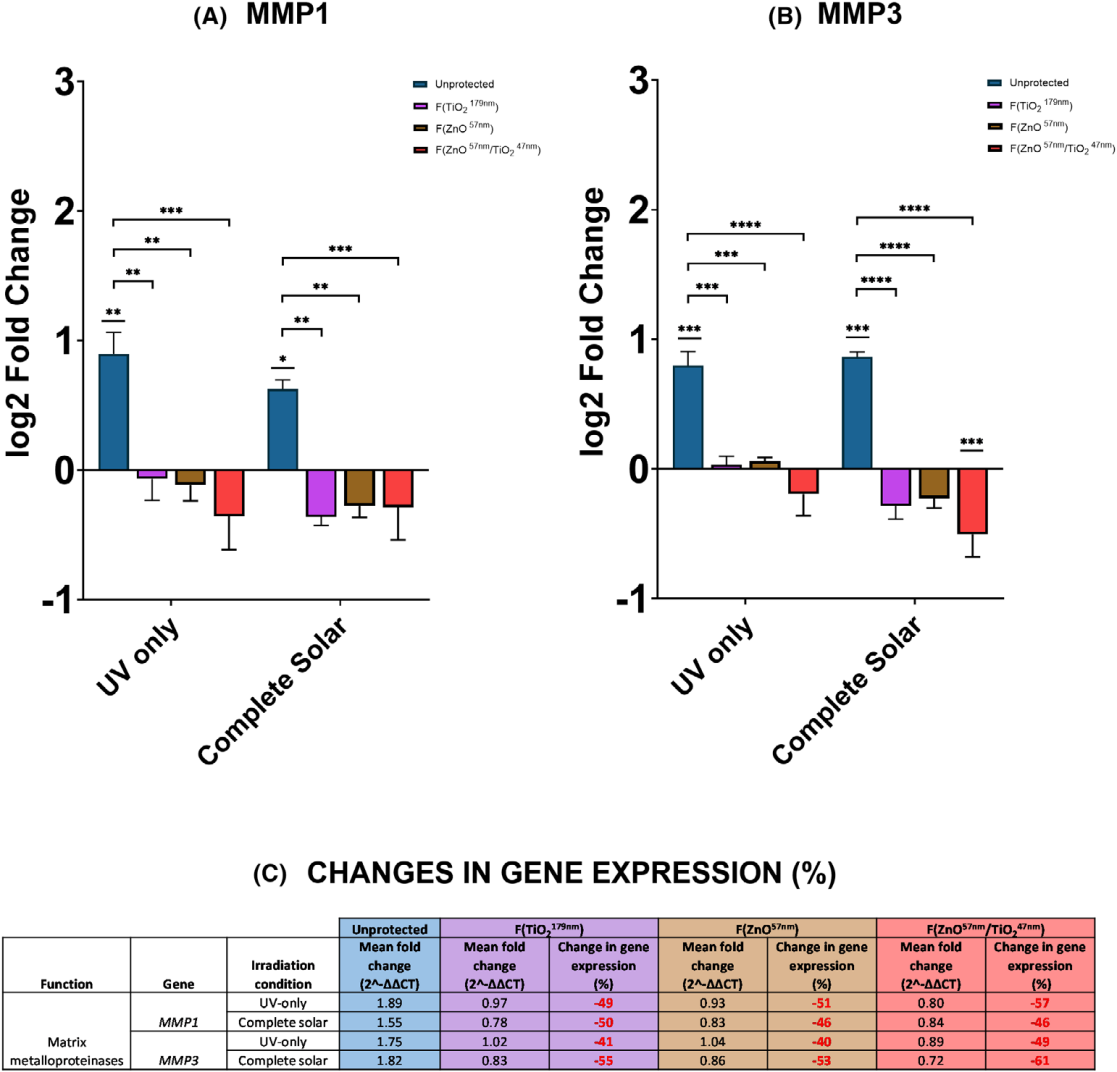
Gene expression of MMP1 and MMP3 in formulation‐protected neonatal human dermal fibroblasts, 24 h after irradiation with UV‐only and complete solar light. Blue bars indicate gene expression in fibroblasts irradiated without photoprotection. Purple bars indicate gene expression in fibroblasts irradiated with F(TiO_2_^179nm^) photoprotection. F(TiO_2_^179nm^) includes titanium dioxide at 7.35% active and is primarily a UVB filter. Brown bars indicate gene expression in fibroblasts irradiated with F(ZnO^57nm^) photoprotection. F(ZnO^57nm^) includes zinc oxide at 25% active and is primarily a UVA filter. Red bars indicate gene expression in fibroblasts irradiated with F(ZnO^57nm^/TiO_2_^47nm^) combined photoprotection. F(ZnO^57nm^/TiO_2_^47nm^) includes zinc oxide and titanium dioxide nanoparticles at 21.4% and 2.4% active, respectively, and is a broad UVR and blue light filter. Fold changes were calculated using the ∆∆Ct method and converted to log_2_ values as shown in the graphs. Percentage differences in gene expression for unprotected against protected fibroblasts are shown in the table below the graphs: Red % change = reduction in expression. Original 2^−ΔΔCt^ mean fold change values were used for percentage change calculations. Expression assayed with TaqMan® qPCR. Data represent mean ± SEM, *n* = 3. *p ≤ 0.05, **p ≤ 0.01, ***p≤ 0.001, ****p ≤ 0.0001.

The three formulations minimized irradiation‐induced changes to MMP1 and MMP3 gene expression by 40–61% (Figure 2). MMP1 was significantly upregulated by both UV‐only and complete solar light in unprotected fibroblasts (UV‐only *p* ≤ 0.01, complete solar *p* ≤ 0.05). Upon applying the formulations, the directionality of MMP1 changed from upregulation to downregulation regardless of irradiation condition and type of formulation. In all protected conditions, MMP1 expression was significantly different compared with the unprotected control (*p* ≤ 0.01) 24 h postirradiation with UV‐only and complete solar light. F(ZnO^57nm^/TiO_2_^47nm^) exhibited the greatest reduction in MMP1 expression (57%), and the greatest magnitude of downregulation (2^−ΔΔCT^ = 0.8; log_2_ foldchange = −0.32) in the UV‐only condition. For complete solar, F(TiO_2_^179nm^) exhibited the greatest alleviation. However, in either irradiation condition, there were no statistical differences between the three formulations in the extent of MMP1 reduction. MMP1 expression in protected HDFns consistently displayed no significant difference against a nonirradiated control.

MMP3 was also significantly upregulated in unprotected fibroblasts following UV‐only and complete solar irradiation (*p* ≤ 0.001). Upon applying the formulations, MMP3 expression generally leaned toward downregulation, although F(TiO_2_^179nm^) and F(ZnO^57nm^) showed weak upregulation close to nonirradiated levels in the UV‐only condition. A key observation is that the complete solar condition led to consistent and greater magnitudes of downregulation, and therefore increased MMP3 reduction, compared with UV‐only. MMP3 reduction in UV‐only was between 40 and 49%, while for complete solar, reduction was between 53 and 61%. In both irradiation conditions, the inorganic‐only (ZnO + TiO_2_) formulation F(ZnO^57nm^/TiO_2_^47nm^) displayed the greatest magnitude of downregulation. Although no statistical differences were found between the three formulations in MMP3 expression, F(ZnO^57nm^/TiO_2_^47nm^) caused a significant downregulation against the nonirradiated control (*p* ≤ 0.001).

#### TiO_2_ , ZnO, and ZnO + TiO_2_ formulations can alleviate irradiation‐induced PTGS1 and PTGES upregulation to below nonirradiated levels

**FIGURE 3:**
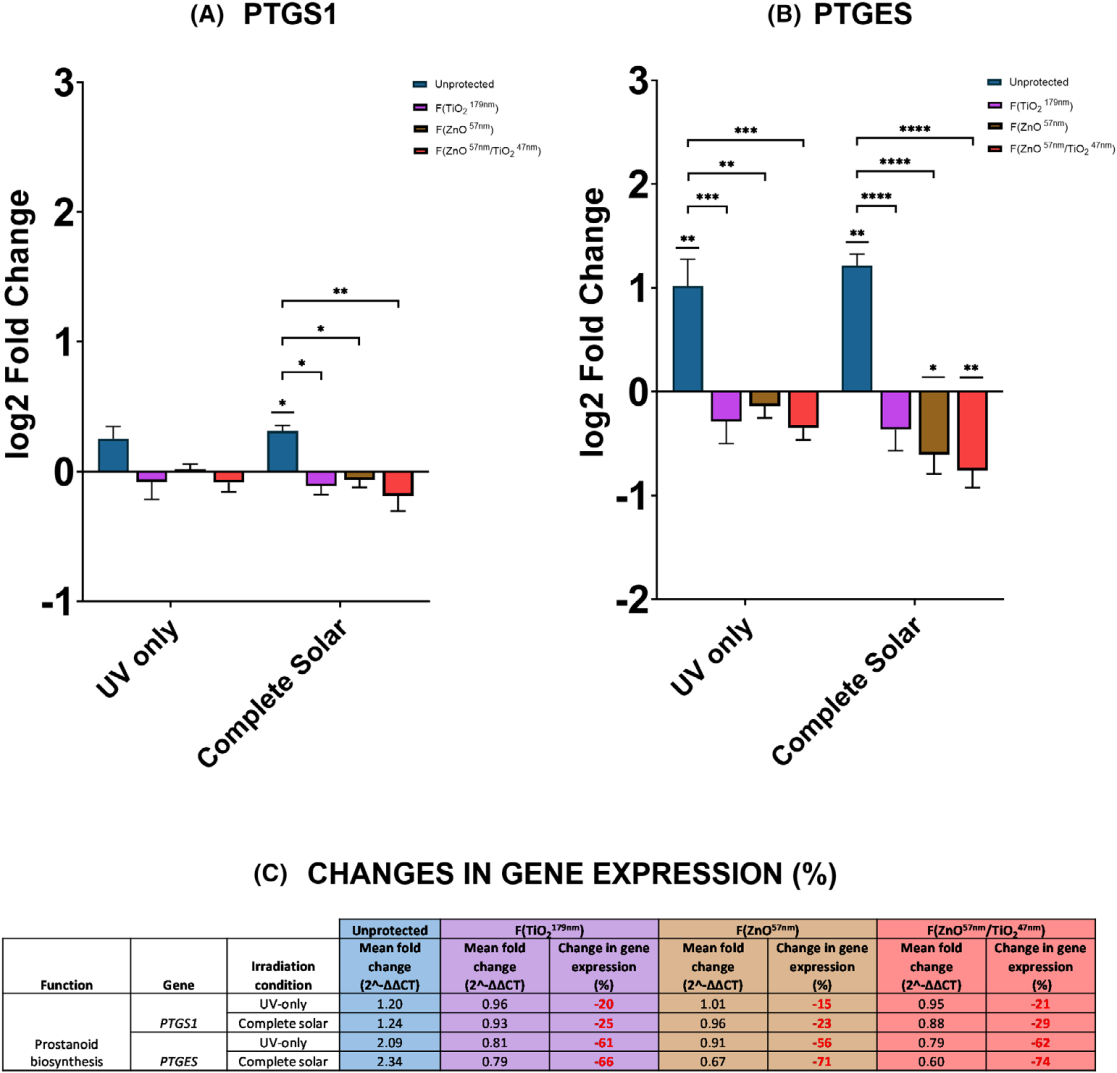
Gene expression of PTGS1 and PTGES in formulation‐protected neonatal human dermal fibroblasts, 24 h after irradiation with UV‐only and complete solar light. Blue bars indicate gene expression in fibroblasts irradiated without photoprotection. Purple bars indicate gene expression in fibroblasts irradiated with F(TiO_2_^179nm^) photoprotection. F(TiO_2_^179nm^) includes titanium dioxide at 7.35% active and is primarily a UVB filter. Brown bars indicate gene expression in fibroblasts irradiated with F(ZnO^57nm^) photoprotection. F(ZnO^57nm^) includes zinc oxide at 25% active and is primarily a UVA filter. Red bars indicate gene expression in fibroblasts irradiated with F(ZnO^57nm^/TiO_2_^47nm^) combined photoprotection. F(ZnO^57nm^/TiO_2_^47nm^) includes zinc oxide and titanium dioxide nanoparticles at 21.4% and 2.4% active, respectively, and is a broad UVR and blue light filter. Fold changes were calculated using the ∆∆Ct method and converted to log_2_ values as shown in the graphs. Percentage differences in gene expression for unprotected against protected fibroblasts are shown in the table below the graphs: Red % change = reduction in expression. Original 2^−ΔΔCT^ mean fold change values were used for percentage change calculations. Expression assayed with TaqMan® qPCR. Data represent mean ± SEM, *n* = 3. *p ≤ 0.05, **p ≤ 0.01, ***p ≤ 0.001, ****p ≤ 0.0001.

Irradiation‐induced expression of the prostanoid biosynthesis genes, PTGS1, and PTGES, was alleviated by all three sunscreen formulations (Figure 3). In unprotected fibroblasts, both genes were upregulated to varying extents. PTGS1 did not exhibit a significant change in gene expression following UV‐only irradiation, and only a low significance was observed in the complete solar condition (*p* ≤ 0.05). However, PTGES was upregulated >2‐fold (log_2_ fold change > 1) in both irradiation conditions (*p* ≤ 0.01). Following protection with F(TiO_2_^179nm^), F(ZnO^57nm^), and F(ZnO^57nm^/TiO_2_^47nm^), upregulation of PTGS1 and PTGES was minimized by 15–29% and 56–74%, respectively.

The three formulations generally caused a change in PTGS1 and PTGES directionality from upregulation to downregulation. The only exception is the impact of F(ZnO^57nm^) on PTGS1 following UV‐only irradiation, which yields an expression close to nonirradiated levels but has a mean value that slightly aligns toward upregulation. Regardless of irradiation condition, F(ZnO^57nm^/TiO_2_^47nm^) is the most impactful formulation for both PTGS1 and PTGES. For the former gene, F(ZnO^57nm^/TiO_2_^47nm^) induces a 21% reduction following UV‐only and a 29% reduction following complete solar irradiation. For the latter gene, F(ZnO^57nm^/TiO_2_^47nm^) induces a 62% reduction following UV‐only and 74% reduction following complete solar irradiation. Similar comparisons can be made with the other two formulations whereby PTGES was alleviated by a greater percentage compared with PTGS1, and the complete solar condition induced greater downregulation than UV‐only.

No statistical differences were observed between the three formulations for PTGS1 and PTGES expression, regardless of irradiation condition. Against a nonirradiated control, PTGS1 expression in protected HDFn was not significantly different in both UV‐only and complete solar conditions. While PTGES expression in protected HDFns was not significantly different against the nonirradiated control in the UV‐only condition, F(ZnO^57nm^) and F(ZnO^57nm^/TiO_2_^47nm^) were statistically different in the complete solar condition (F(ZnO^57nm^): *p* ≤ 0.05; F(ZnO^57nm^/TiO_2_^47nm^): *p* ≤ 0.01).

#### TiO_2_ , ZnO, and ZnO + TiO_2_ formulations significantly reduce irradiation‐induced changes in most cell cycle‐related genes

**FIGURE 4:**
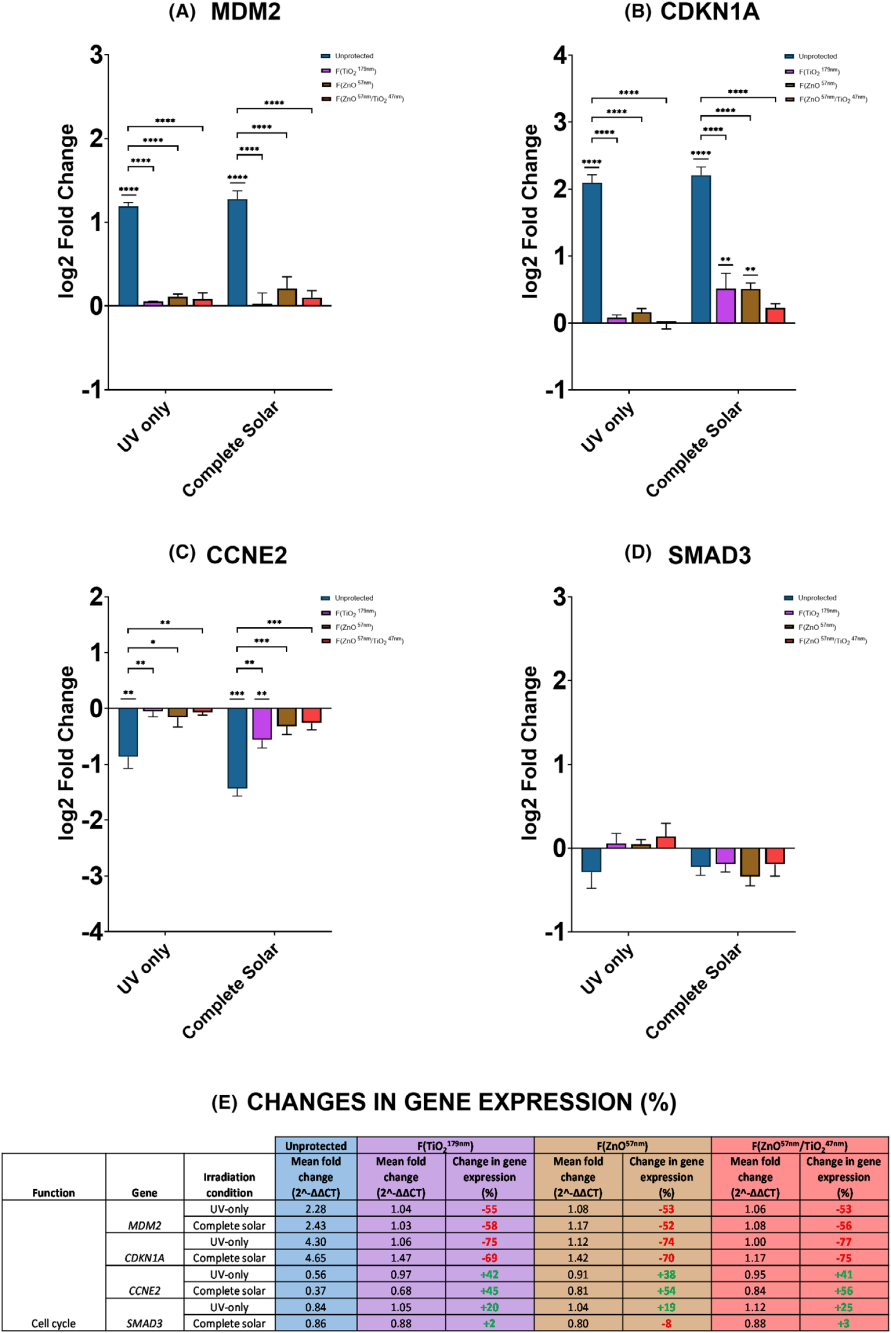
Gene expression of MDM2, CDKN1A, CCNE2, and SMAD3 in formulation‐protected neonatal human dermal fibroblasts, 24 h after irradiation with UV‐only and complete solar light. Blue bars indicate gene expression in fibroblasts irradiated without photoprotection. Purple bars indicate gene expression in fibroblasts irradiated with F(TiO_2_^179nm^) photoprotection. F(TiO_2_^179nm^) includes titanium dioxide at 7.35% active and is primarily a UVB filter. Brown bars indicate gene expression in fibroblasts irradiated with F(ZnO^57nm^) photoprotection. F(ZnO^57nm^) includes zinc oxide at 25% active and is primarily a UVA filter. Red bars indicate gene expression in fibroblasts irradiated with F(ZnO^57nm^/TiO_2_^47nm^) combined photoprotection. F(ZnO^57nm^/TiO_2_^47nm^) includes zinc oxide and titanium dioxide nanoparticles at 21.4% and 2.4% active, respectively, and is a broad UVR and blue light filter. Fold changes were calculated using the ∆∆Ct method and converted to log_2_ values as shown in the graphs. Percentage differences in gene expression for unprotected against protected fibroblasts are shown in the table below the graphs: Red % change = reduction in expression. Original 2^−ΔΔCT^ mean fold change values were used for percentage change calculations. Expression assayed with TaqMan® qPCR. Data represent mean + ‐SEM, *n* = 3. *p ≤ 0.05, **p ≤ 0.01, ***p ≤ 0.001, ****p ≤ 0.0001.

Irradiation‐induced changes in three of the four cell cycle genes investigated were significantly alleviated upon protection with the three formulations (Figure 4). The three genes which are significantly impacted are MDM2, CDKN1A, and CCNE2. Upregulation of MDM2 and CDKN1A, as well as downregulation of CCNE2, caused by UV‐only and complete solar irradiation were all significantly minimized upon protection with TiO_2_^47nm^, ZnO^57nm^, and TiO_2_^179nm^. In unprotected fibroblasts, MDM2 and CDKN1A were significantly upregulated by UV‐only and complete solar light (*p* ≤ 0.0001). Furthermore, both genes were alleviated 52–58% and 69–77%, respectively, following protection with any of the three sunscreen formulations. Both genes exhibited some of the greatest differences in fold change between unprotected and protected fibroblasts among all 11 marker genes investigated, with CDKN1A being the most impacted. The significance in difference for these two genes was indiscriminate of the type of formulation applied and the irradiation condition (*p* ≤ 0.0001).

Directionality of MDM2 and CDKN1A was preserved; expression remained upregulated despite significant reductions in irradiation‐induced expression. For MDM2, expression was reduced to levels that are not significantly different from the nonirradiated control in UV‐only and complete solar light with all three formulations. On the contrary, for CDKN1A, this effect was only visible in the UV‐only condition. F(TiO_2_^179nm^) and F(ZnO^57nm^) reduced CDKN1A expression by 69% and 70%, respectively, in the complete solar condition but remained significantly different from CDKN1A expression in nonirradiated conditions (*p* ≤ 0.01). Regardless, for both MDM2 and CDKN1A, no statistical differences were observed when comparing the extent of alleviation between the three formulations.

CCNE2 was significantly downregulated in unprotected fibroblasts upon irradiation with UV‐only (*p* ≤ 0.01) and complete solar light (*p* ≤ 0.001). The three formulations promisingly reduced the extent of downregulation by 38–56% but maintained directionality. All formulations caused an increase in CCNE2 that narrowed the difference between irradiated and nonirradiated expression to nonsignificant levels in the UV‐only condition. As for the complete solar condition, this only occurred with F(ZnO^57nm^) and F(ZnO^57nm^/TiO_2_^47nm^). Although a 45% increase in CCNE2 was observed with F(TiO_2_^179nm^) following complete solar irradiation, the final expression value (2^−ΔΔCT^ = 0.68; log_2_ foldchange = −0.56) was still significantly different against a nonirradiated control (*p* ≤ 0.01).

In the UV‐only condition, CCNE2 expression increased by 45, 40, and 44% following protection with TiO_2_^47nm^, ZnO^57nm^, and TiO_2_^179nm^, respectively. Likewise, in the complete solar condition, CCNE2 expression increased by 56, 54, and 61%, respectively. CCNE2 expression in the complete solar condition remained significantly different from a nonirradiated control with TiO_2_^47nm^ and ZnO^57nm^ protection (*p* ≤ 0.05). The directionality of expression was conserved regardless of UV filter and irradiation condition; CCNE2 remained downregulated. There were no significant differences in gene expression between the three UV filters (*p* ≥ 0.05).

The irradiation‐induced expression of the final cell cycle gene, SMAD3, was generally alleviated by the three formulations. The increase in SMAD3 upon protection with formulations was between 2 and 20%. However, F(ZnO^57nm^) was an outlier as it appeared to aggravate the downregulation caused by complete solar irradiation, causing an 8% increase in irradiation‐induced downregulation instead. Regardless, no significant differences were observed between protected and unprotected fibroblasts. Likewise, no significant differences were observed between any of the conditions and the nonirradiated control. Compared with the other cell cycle genes, there were no trends that could be considered statistically different.

### Protein Analyses of MMP1, PTGES, and p21 (CDKN1A)

#### Protein analyses of MMP1 24 h postirradiation with 2.16 SED UV‐only and complete solar light

**FIGURE 5:**
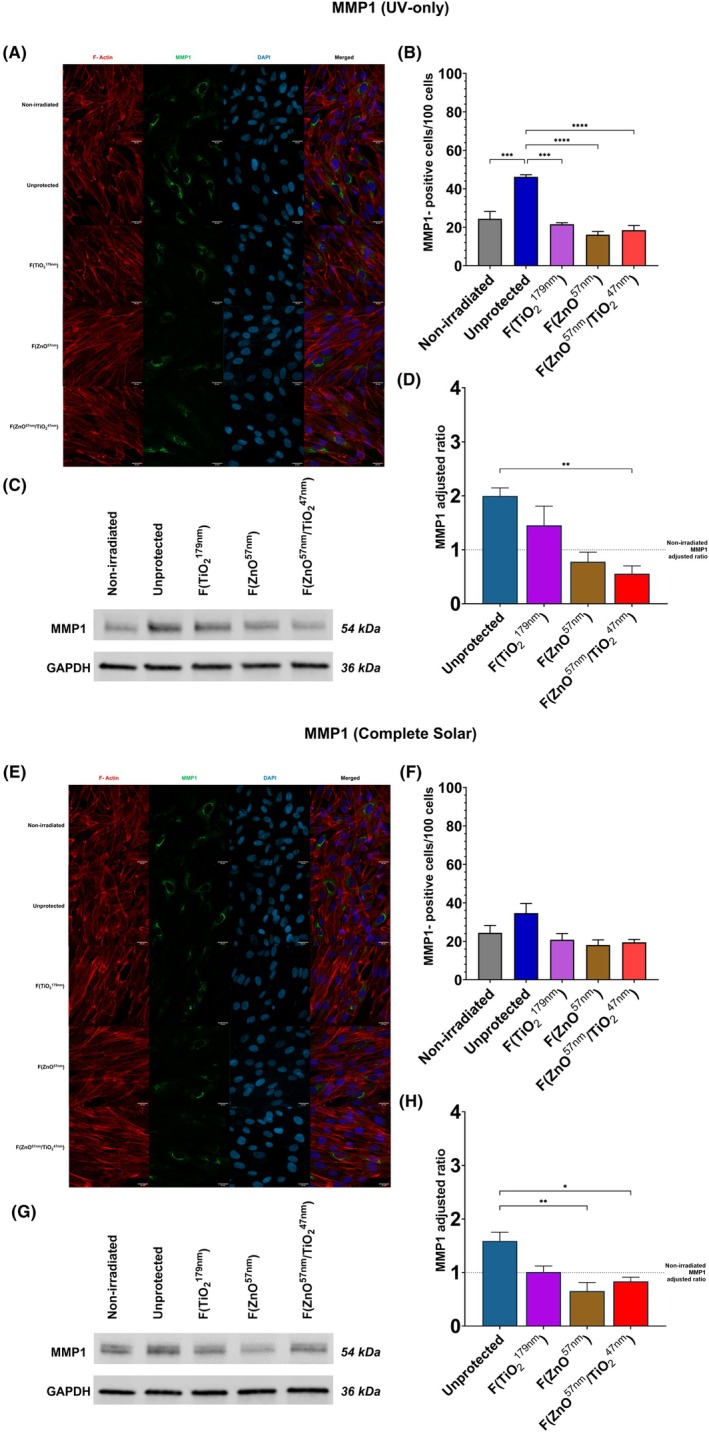
Protein analyses of MMP1 in HDFn 24 h postirradiation with UV‐only and complete solar light. Immunocytochemistry (ICC) and Western blotting (WB) showed that MMP1 expression was lower in formulation‐protected human neonatal dermal fibroblast (HDFn) compared with unprotected HDFn, in response to UV‐only (A–D) and complete solar light (E–H). HDFn were labeled with MMP1 polyclonal antibody (Catalog #PA5‐27210) for both analyses, at 5 μg/mL and 1:500 dilution, respectively. Alexa Fluor™ 488‐conjugated goat anti‐rabbit secondary antibody (Catalog # A11008) at 1:500 dilution was used for ICC, while Goat anti‐rabbit IgG (H + L) cross‐adsorbed secondary antibody, Alexa Fluor™ Plus 488 (Catalog # A‐11008), at 1:1000 dilution was used for WB. (A and E) ICC qualitative analysis. F‐actin was observed with Alexa Fluor™ 555 Phalloidin (Catalog # A34055) at 1:400 dilution and DAPI was observed with Prolong™ Diamond Antifade Mountant with DAPI (Catalog # P36962). Scale: 20 μm. (B and F). ICC quantitative analysis. (C and G) Representative WB images comparing MMP1 bands at 54 kDa to a loading control GAPDH at 36 kDa. GAPDH was probed separately using GAPDH polyclonal antibody (FL‐335) (Catalog # sc‐25778) at 1:2000 dilution. (D and H) Intensity values for MMP1 bands were normalized to GAPDH and adjusted as a ratio to the nonirradiated control using densitometric analysis. Data represent mean ± SEM, *n* = 3. *p ≤ 0.05, **p ≤ 0.01, ***p ≤ 0.001, ****p ≤ 0.0001.

Immunofluorescence analysis of MMP1 shows that MMP1 is detected in the cytoplasm, and not in the nucleus of HDFn (Figure 5A,E). By counterstaining with F‐actin and DAPI, MMP1 expression is observed not to spread equally across the cytoplasm and tends to be localized close to the edges of the nucleus. There, MMP1 emits a stronger fluorescent signal which is detected by the 488 nm laser of the confocal microscope. By contrasting the number of nuclei to the fluorescent stains for MMP1, as seen in the merged image, only some cells express detectable MMP1. Thus, quantification of MMP1 was possible by quantifying the number of MMP1‐positive cells in a cell population (Figure 5B,F).

In the nonirradiated condition, 24% of HDFn were MMP1‐positive. The percentage of the MMP1 population increased 24 h postirradiation with 2.16 SED UV‐only to 46%; this indicates a near doubling of MMP1 expression (Figure 5B). The difference between the nonirradiated and unprotected conditions exhibited a strong significant difference (*p* ≤ 0.001). On the contrary, complete solar light increased the population of MMP1‐positive cells by 10%, to a total of 34% of the population (Figure 5F). However, no significance was observed.

Application of the three formulations during irradiation reduced the MMP1‐positive population from unprotected levels to below nonirradiated levels. In the UV‐only condition, the three formulations caused a 50% or greater reduction in MMP1‐positive cells compared with unprotected HDFns. F(ZnO^57nm^) was most effective, reducing the MMP1‐positive population to 15%. On the contrary, the three formulations caused a maximum of 34% decrease in the MMP1‐positive population from unprotected levels following complete solar light irradiation. Likewise, F(ZnO^57nm^) yielded the lowest MMP1‐positive population at 18%. In both irradiation conditions, no significant differences were observed between the three formulations in terms of reducing MMP1.

Western blot results demonstrated similar results to immunocytochemistry whereby unprotected HDFn express a higher concentration of MMP1 compared with the nonirradiated condition and the formulation‐protected conditions (Figure 5C,D,G,H). The antibody used to detect MMP1 showed affinity for two glycosylated forms of MMP1 close to 54 kDa; this materialized as two individual bands that were on top of one another in the same region of the nitrocellulose membrane. At 24 h, unprotected HDFn consistently exhibited bands with stronger intensity following irradiation with 2.16 SED UV‐only and complete solar light (Figure 5C,G). Densitometric analysis showed that like immunocytochemistry, MMP1 expression in unprotected fibroblasts was greater in UV‐only than solar light. Unprotected fibroblasts displayed a ~2‐fold (100%) increase in MMP1 following UV‐only irradiation compared with nonirradiated conditions (Figure 5D). Alternatively, unprotected fibroblasts had a ~1.6‐fold (60%) increase in MMP1 following complete solar irradiation (Figure 5H).

Upon applying the three formulations, the MMP1 bands displayed lighter intensity, although varying. As supported by the densitometric analysis, this inferred reduced expression of MMP1 in all formulation‐protected fibroblasts. In UV‐only, F(ZnO^57nm^/TiO_2_^47nm^) was observed to have the least expression of MMP1 with a mean ratio of 0.557; in other words, a 45% decrease from nonirradiated fibroblasts or a 72% decrease from unprotected fibroblasts (Figure 5D). In complete solar, F(ZnO^57nm^) had the least expression of MMP1, with a mean ratio of 0.654 that translates to a 35% decrease from nonirradiated fibroblasts and a 59% decrease from unprotected fibroblasts (Figure 5H). In both irradiation conditions, a significant difference in MMP1 expression against unprotected HDFn was identified for F(ZnO^57nm^/TiO_2_^47nm^; UV‐only: *p* ≤ 0.01; Complete Solar: *p* ≤ 0.05). MMP1 expression with F(ZnO^57nm^) was significantly different from that in unprotected HDFn (*p* ≤ 0.05), exclusively in the complete solar condition.

#### Protein analyses of PTGES 24 hours postirradiation with 2.16 SED UV‐only and complete solar light

**FIGURE 6:**
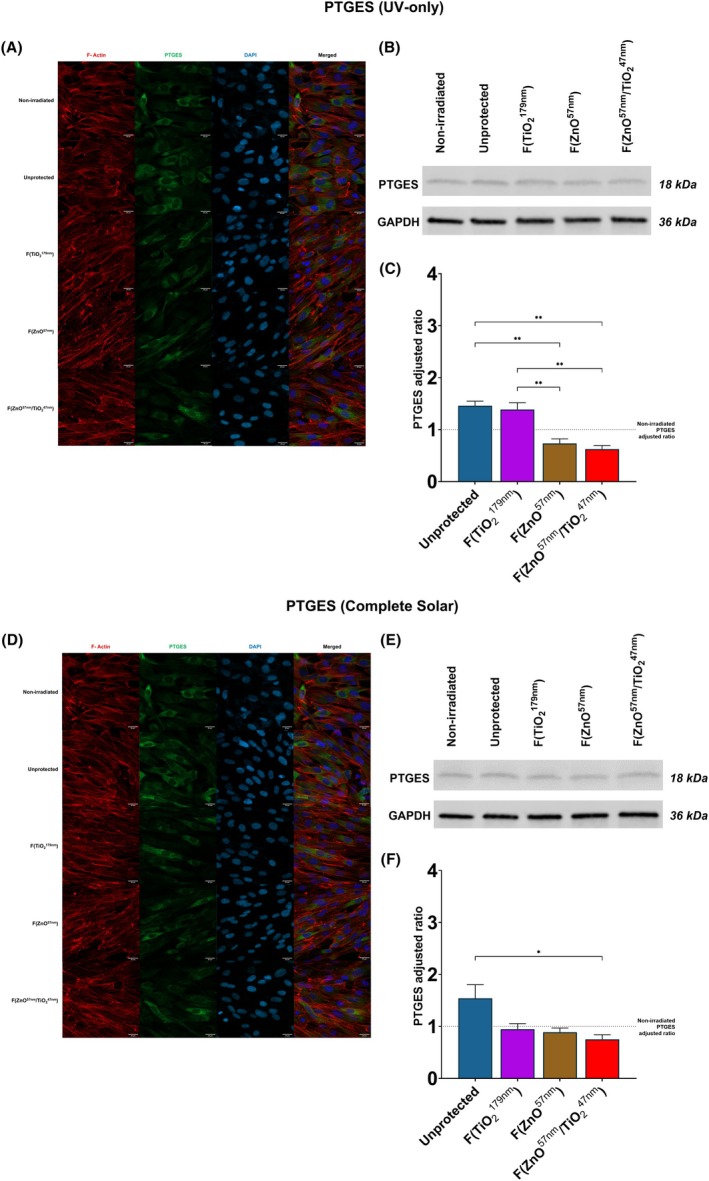
Protein analyses of PTGES in HDFn 24 h postirradiation with UV‐only and complete solar light. Immunocytochemistry (ICC) and Western blotting (WB) showed that PTGES expression was lower in formulation‐protected human neonatal dermal fibroblast (HDFn) compared with unprotected HDFn, in response to UV‐only (A–C) and complete solar light (D–F). HDFn were labeled with PTGES polyclonal antibody (Catalog #PA5‐60916) for both analyses, at 5 μg/mL and 1:1000 dilution, respectively. Alexa Fluor™ 488‐conjugated goat anti‐rabbit secondary antibody (Catalog # A11008) at 1:500 dilution was used for ICC, while Goat anti‐rabbit IgG (H + L) cross‐adsorbed secondary antibody, Alexa Fluor™ Plus 488 (Catalog # A‐11008), at 1:1000 dilution was used for WB. (A and D) ICC qualitative analyses. F‐actin was observed with Alexa Fluor™ 555 Phalloidin (Catalog # A34055) at 1:400 dilution and DAPI was observed with Prolong™ Diamond Antifade Mountant with DAPI (Catalog # P36962). Scale: 20 μm. (B and E). Representative WB images comparing PTGES bands at 18 kDa to a loading control GAPDH at 36 kDa. GAPDH was probed separately using GAPDH polyclonal antibody (FL‐335) (Catalog # sc‐25778) at 1:2000 dilution. (C and F) Intensity values for PTGES bands were normalized to GAPDH and adjusted as a ratio to the nonirradiated control using densitometric analysis. Data represent mean ± SEM, *n* = 3. *p ≤ 0.05, **p ≤0.01.

Immunofluorescence analysis of PTGES detected in the cytoplasm and not in the nucleus of HDFn (Figure 6A,D). Counterstaining with F‐actin shows that PTGES is expressed across the entire cytoplasm of the fibroblast; it is not limited to a certain area like MMP1. PTGES was detected in most cells, although to varying levels of fluorescence detected by the 488 nm laser of the confocal microscope. While fluorescence was observable in some cells, it appeared faint in others, particularly in cells which have been overlapped by another in the same plane of view. The main observable difference in PTGES expression was that more fibroblasts exhibited increased fluorescence in a single plane of view in the unprotected condition. Despite similar cell populations, a single plane of view in the nonirradiated condition and formulation‐protected fibroblasts yielded fewer cells of equal fluorescence.

Due to the broad area of PTGES localization, fluorescence detection in most cells, and the overlapping nature of HDFns in monolayer culture, it was not possible to accurately quantify the images without bias, due to several parameters such as cell shape and area, confluency, and plane of view. To maintain consistency with other results, none of these parameters were altered. Thus, quantification was only conducted with Western blotting.

Western blot results found that UV‐only and complete solar irradiation caused PTGES expression to increase by 46 and 54% (Figure 6B,C,E,F). The antibody used to detect PTGES bound to the protein at 18 kDa and appeared as a single band. At 24 h postirradiation with UV‐only and complete solar light, unprotected HDFn consistently exhibited bands with faint intensity relative to the loading control GAPDH (Figure 6B,E). Densitometric analysis however showed that there were variances in the intensity of the bands between sample conditions.

The UV‐only condition showed that F(ZnO^57nm^) and F(ZnO^57nm^/TiO_2_^47nm^) alleviated the irradiation‐induced increase in PTGES (Figure 6C). The formulations induced a 50% and 57% reduction, respectively. Furthermore, PTGES expression with these two formulations led to a PTGES adjusted ratio that was lower than the nonirradiated control. However, F(TiO_2_^179nm^) did not appear to be effective in reducing irradiation‐induced PTGES expression, as there was only a 5% reduction, and PTGES remained elevated. PTGES expressions for both unprotected fibroblasts and F(TiO_2_^179nm^) were significantly different from F(ZnO^57nm^) and F(ZnO^57nm^/TiO_2_^47nm^; *p* ≤ 0.01).

Meanwhile, the complete solar condition showed that all three formulations caused >35% decrease in PTGES expression (Figure 6F). F(ZnO^57nm^/TiO_2_^47nm^) alleviated PTGES to a greater extent than the other two formulations, with a mean value of 0.752 indicating a 51% decrease from unprotected fibroblasts and a 25% decrease from nonirradiated fibroblasts. For all formulations, PTGES expression was reduced to below nonirradiated levels. A statistical significance in PTGES expression for complete solar light was only observed between the unprotected condition and F(ZnO^57nm^/TiO_2_^47nm^; *p* ≤ 0.05).

#### Protein analyses of p21 24 h postirradiation with 2.16 SED UV‐only and complete solar light

**FIGURE 7:**
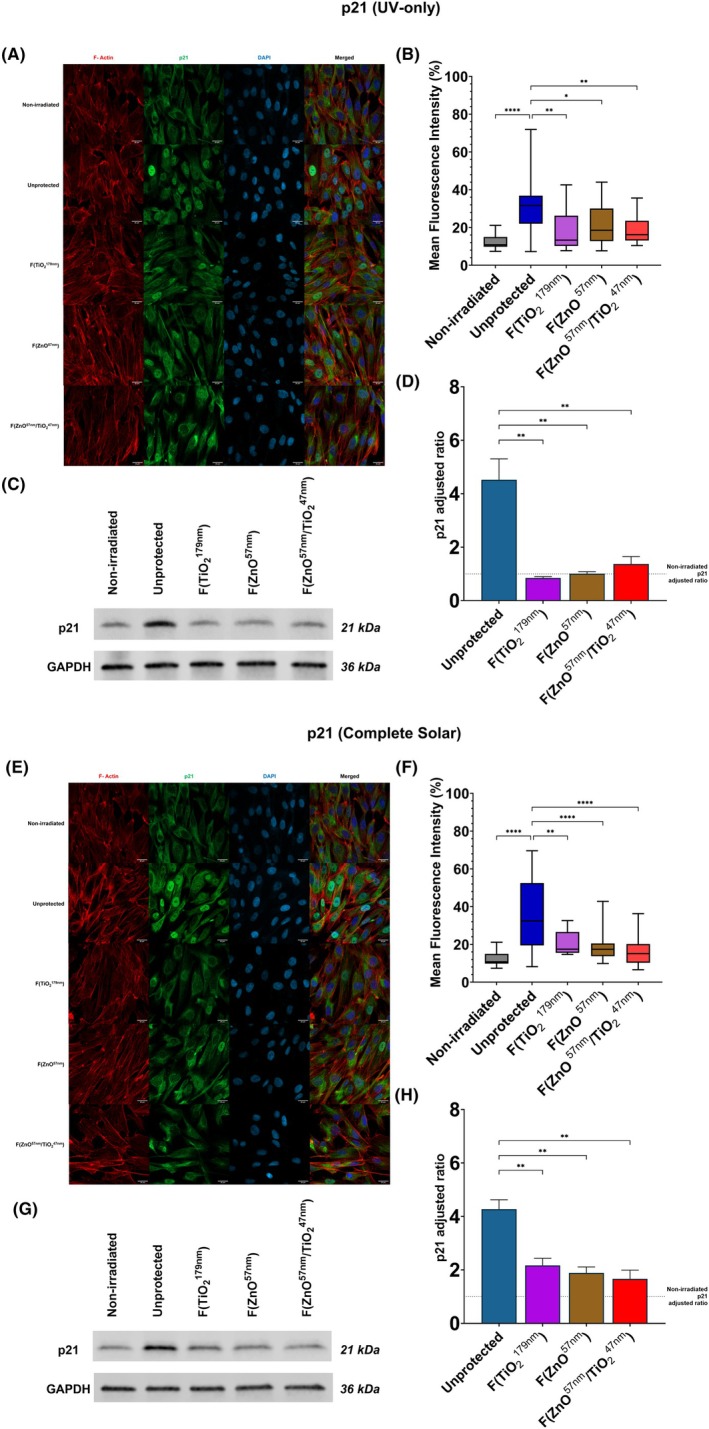
Protein analyses of p21 in HDFn 24 h postirradiation with UV‐only and complete solar light. Immunocytochemistry (ICC) and Western blotting (WB) showed that p21 expression was lower in formulation‐protected human neonatal dermal fibroblast (HDFn) compared with unprotected HDFn, in response to UV‐only (A–D) and complete solar light (E–H). HDFn were labeled with p21 polyclonal antibody (Catalog #10355‐1‐AP) for both analyses, at 1:100 and 1:1000 dilution, respectively. Alexa Fluor™ 488‐conjugated goat anti‐rabbit secondary antibody (Catalog # A11008) at 1:500 dilution was used for ICC, while Goat anti‐rabbit IgG (H + L) cross‐adsorbed secondary antibody, Alexa Fluor™ Plus 488 (Catalog # A‐11008), at 1:1000 dilution was used for WB. (A and E) ICC qualitative analyses. F‐actin was observed with Alexa Fluor™ 555 Phalloidin (Catalog # A34055) at 1:400 dilution and DAPI was observed with Prolong™ Diamond Antifade Mountant with DAPI (Catalog # P36962). Scale: 20 μm. (B and F) ICC quantitative analysis. (C and G) Representative WB images comparing p21 bands at 21 kDa to a loading control GAPDH at 36 kDa. GAPDH was probed separately using GAPDH polyclonal antibody (FL‐335) (Catalog # sc‐25778) at 1:2000 dilution. (D and H) Intensity values for p21 bands were normalized to GAPDH and adjusted as a ratio to the nonirradiated control using densitometric analysis. Data represent mean ± SEM, *n* = 3. *p ≤ 0.05, **p ≤ 0.01, ****p ≤ 0.0001.

Immunofluorescence analysis of p21 shows that it is detected in both the nucleus and cytoplasm (Figure 7A,E). Upon counterstaining with F‐actin, p21 was observed to be localized broadly across the cytoplasm like PTGES. Additionally, upon counterstaining with DAPI, p21 was found to be localized in the nucleus as tiny but abundant specks. While the fluorescence of cytoplasmic p21 detected by the 488 nm laser of the confocal microscope was consistent per cell and per condition, nuclear p21 was variable.

In nonirradiated HDFn, p21 fluorescence was characterized as abundant fluorescent specks encased within the nucleus. However, in irradiated and unprotected HDFn, the nucleus had both specks and a halo of fluorescent signal which encompassed the entire nucleus. It is worth noting that nuclear p21 fluorescence varied broadly from cell to cell in each sample condition. Some cells had specks with no nuclear background fluorescence while others exhibited maximum nuclear background fluorescence, whereby specks could no longer be observed with the resolution used. The number of specks in each nucleus was primarily dependent on the size of the nucleus rather than the irradiation condition or sample condition. However, the nuclear background fluorescence was impacted by UV‐only and complete solar light. In the unprotected condition, it was observed that there were a greater number of cells with nuclei that exhibited nuclear fluorescent background and increased fluorescence intensity.

Upon applying the three inorganic formulations, the number of fibroblasts that exhibited nuclear background fluorescence decreased. Furthermore, an increased number of fibroblasts only exhibited specks of p21 in the nucleus. This suggests that the formulations reduced nuclear p21 expression but not to nonirradiated levels. Quantitative measurements were taken to support the observable results by measuring the mean fluorescence intensity of each nucleus.

Box plots were produced which showed that the mean fluorescence intensity for nonirradiated HDFns was 10–11%, while for unprotected HDFns it was 32–33%. The mean fluorescence intensities for these two sample conditions were similar for UV‐only and complete solar (Figure 7B,F). In contrast, the mean fluorescence intensities for F(TiO_2_^179nm^), F(ZnO^57nm^), and F(ZnO^57nm^/TiO_2_^47nm^) were 14, 19, and 17% for UV‐only, and 17, 17, and 15%, respectively. From these values, there was only a 5% margin of difference between the three formulations and thus no significant differences were observed. However, a significance in mean fluorescence intensity was observed between the unprotected condition and other sample conditions, in both UV‐only and complete solar irradiation conditions (UV‐only: *p* ≤ 0.05; Complete Solar: *p* ≤ 0.01).

The box plots also provide additional information that describes the extent of p21 activation in a fibroblast population. The minimum fluorescence intensity of nonirradiated fibroblasts in either irradiation condition was 6–7%. In UV‐only, a similar minimum fluorescence intensity was observed with F(TiO_2_^179nm^) and (ZnO^57nm^) protection; in complete solar, this was observed with F(ZnO^57nm^/TiO_2_^47nm^) protection. Based on these two observations, it can be suggested that p21 does not undergo UV‐based activation in every fibroblast at 24 h, regardless of protection or lack thereof. Another box plot observation is the wide range of fluorescence intensities indicating inequality in p21 activation within the fibroblast population. This was prominent in the unprotected condition, which had a minimum of 7% and maximum of 71% in UV‐only, and a minimum of 7% and maximum of 69% in complete solar.

Western blot analysis contributed to the quantified trends observed in the box plots (Figure 7C,D,G,H). The antibody used to detect p21 bound to the protein at 21 kDa and appeared as a single band. However, the antibody also nonspecifically bound between 58 and 62 kDa. At 24 h postirradiation with UV‐only and complete solar light, unprotected HDFn consistently exhibited bands with strong intensity relative to the other sample conditions, which produced bands that were significantly lighter in intensity (Figure 7C,G). The density of p21 bands in the unprotected conditions appeared almost similar in intensity to the loading control GAPDH.

Densitometric analysis showed that 2.16 SED UV‐only irradiation caused an increase in the p21 adjusted ratio to 4.52 in unprotected fibroblasts after 24 h; there was a 452% increase in p21 expression compared with the nonirradiated control (Figure 7D). Similarly, a 427% increase was observed in the complete solar condition (Figure 7H). Upon application of the three inorganic formulations, irradiation‐induced p21 elevation was significantly minimized, especially in the UV‐only condition. It was found that p21 expression was reduced close to nonirradiated levels (±36%). While p21 remained elevated by 1.6–2.2‐fold, despite protection in the complete solar condition, a significant reduction of up to 61% was still evident (*p* ≤ 0.01). There were no significant differences observed between the three formulations regarding the extent of p21 reduction; however, the formulation with the greatest impact varied depending on the irradiation condition. F(TiO_2_^179nm^) was most effective against UV‐only while F(ZnO^57nm^/TiO_2_^47nm^) was most effective for complete solar. Regardless, p21 expression with all three formulations was statistically different (*p* ≤ 0.01).

## DISCUSSION

### Formulations must be developed with regards to function and cosmetic appeal

The formulations developed in this study were based on existing commercial sunscreen combinations that utilize oil‐based TiO_2_ and ZnO particles. The formulations were O/W formulations, meaning that the oil phase, which includes the inorganic UV filters, is broadly suspended as lipid droplets within a water medium. There are several benefits to using O/W formulations over water‐in‐oil (W/O), including good spreadability and moisturizing feel on application.^29^, ^30^ Glyceryl stearate and sorbitan stearate were used as emulsifiers for the formulations, ensuring that the aqueous phase and oil phase did not separate. Glyceryl stearate was also used for its secondary benefit of being a humectant and emollient; it is well known to display good biocompatibility and retain moisture.^31^, ^32^ Aside from the two stearate‐based emulsifiers, other emulsifiers that have a dual role as thickeners were also present in the formulation, such as Tween 60, stearyl alcohol, xanthan gum, and magnesium aluminum silicate.

Cosmetic appeal is an aspect which must also be considered regarding formulations. TiO_2_ and ZnO particles both display high refractive indexes, which means that they both produce a whitening effect on skin^15^, ^33^; this can be undesirable in various parts of the world. Formulation scientists must therefore consider transparency as a key aesthetic parameter when developing new sunscreens. An approach to minimize whitening is to reduce the size of the particles to <200 nm, ideally to nanoparticle size at around 20 nm, which promotes transparency.^15^, ^34^, ^35^ In this study, the formulations contained TiO_2_ with a mean particle size of 47 nm or 179 nm and ZnO with a mean particle size of 57 nm. F(TiO_2_^179nm^) which contained larger‐sized nanoparticles, appeared considerably whiter than F(ZnO^57nm^) and F(ZnO^57nm^/TiO_2_^179nm^) which both contained nanoparticles when applied on the Transpore tape (Table 2). This confirmed that although all inorganic UV filters were <200 nm, nanoparticle‐sized particles (1–100 nm) provided greater transparency.

However, greater transparency may not necessarily mean greater UVA and UVB protection. As observed in the absolute irradiance spectra in Figure 1, formulations containing the larger particle, TiO_2_^179nm^ blocked more light in the UVB, UVA, and blue light ranges compared with nanoparticle‐based formulations. Although nanoparticles have greater photocatalytic interactions with UV, larger inorganic particles produce a more robust physical barrier that reflects and refracts light more efficiently.^15^, ^36^ This produces a dilemma for formulation scientists who must balance cosmetic appeal and function.

### Formulations alleviate MMP upregulation by preventing ROS‐mediated or direct AP‐1 signaling

MMP1 and MMP3 are genes which code for respective matrix metalloproteinases that degrade ECM proteins. The former is a collagenase which primarily degrades collagen, the major ECM protein that gives the skin structure, while the latter is a stromelysin that has dual functions with ECM remodeling and inflammatory signaling. The upregulation of both genes in unprotected fibroblasts following UVA and UVB exposure correlates to previous observations in the literature.^37^, ^38^, ^39^, ^40^

Photoprotection with the three formulations reduced irradiation‐induced MMP1 gene expression by 46–57%. Similarly, MMP3 was reduced by 40–61% (Figure 2). The bulk of this reduction would be attributed to the inorganic particles broadly blocking UV wavelengths and blue light in the complete solar condition, as observed in the absolute irradiance spectra acquired (Figure 1). At a cellular level, less UV exposure would influence ROS production, MAPK signaling, and AP‐1 activation.^41^, ^42^, ^43^, ^44^ All three have a role in UV stress response that can trigger downstream MMP production and are sensitive to both UVA and UVB.

ROS production in fibroblasts tends to be more prevalent due to UVA.^45^ However, this is applicable only to full‐thickness skin whereby UVB cannot penetrate past the epidermis.^46^ This introduces a limitation to this study whereby 2D monolayers of dermal fibroblasts can be directly exposed to both UVA and UVB. With this limitation in mind, it is possible that the differential gene expression of MMP1 and MMP3 observed is a result of UVA and UVB‐induced ROS production. Increased ROS can in turn activate MAPK signaling, which then activates AP‐1 signaling for MMP production.^44^ However, some studies have shown that UV can directly activate AP‐1 without ROS.^43^ Reduced upregulation of MMP1 and MMP3 upon protection with TiO_2_ and ZnO formulations asserts that direct or indirect stimulation of AP‐1 signaling is prevented, likely from reduced UVA and UVB dose reaching the fibroblasts.

For MMP1, a similar trend was observed with protein expression, as observed by immunocytochemistry, whereby MMP1 alleviation by formulations was greater in the UV‐only condition (Figure 5). There was a maximum difference of 29% in the reduction of MMP1‐positive cells between irradiation conditions. Likewise, Western blot identified that the formulations could reduce MMP1 levels by up to 72% in the UV condition compared with a maximum of 60% in complete solar. Due to the consistency between gene and protein expressions, it can be suggested that decreased alleviation in the complete solar condition may be caused by the inability of TiO_2_ and ZnO particles to effectively block non‐UV wavelengths. This is of concern to MMP1 as blue light wavelengths overlap with UVA and have been reported to induce stress responses like UV.^47^, ^48^ There are various existing studies on skin fibroblasts and keratinocytes which have isolated blue light as a separate light source and found that both gene and protein expression of MMP1 increase with exposure to 420 and 450 nm.^49^, ^50^ It is worth noting that both wavelengths are greater than the overlapping wavelengths with UVA, thus clarifying that MMP1 is impacted by irradiation beyond UV. For blue light, MMP‐1 related photoaging may be caused by activation of the TGF‐β and JNK pathways.^50^

With regards to UV, TiO_2_, and ZnO effectively block UVA and UVB that may trigger MMP1 production. MMP1 production can occur through ROS‐mediated or direct AP‐1 activation and various signaling such as MAPK and JNK.^43^, ^44^ AP‐1 activation, which leads to MMP1 gene activation, occurs in the nucleus. Meanwhile, MMP1 production typically occurs in the cytoplasm, whereby MMP1 protein is first synthesized as a precursor, pro‐MMP1. This precursor is transferred to the endoplasmic reticulum, where its N‐terminal signal sequence is removed to yield MMP1.^51^ The intense fluorescence observed close to the nucleus in immunocytochemistry may be newly synthesized MMP1 being produced in the endoplasmic reticulum, as the organelle is physically connected to the nucleus (Figure 5A,E). Upon synthesis, MMP1 protein can be transported toward the cell membrane and into the ECM, where it can degrade collagen and other ECM proteins; continuous and uncontrolled degradation can lead to photoaging.^52^ Based on the gene expression, immunofluorescence images, and Western blot protein analyses acquired, it can be asserted that the three formulations significantly minimize the sequence of events which lead to UV‐induced ECM protein degradation by MMP1, particularly in the UV‐only condition.

### Formulations alleviate PTGS1 and PTGES upregulation which prevents ROS‐mediated PGE2 production

PTGS1 and PTGES are genes involved in the *prostanoid biosynthesis* pathway, coding for cyclooxygenase‐1 and prostaglandin‐E‐synthase, respectively. The former catalyzes the conversion of arachidonic acid to prostaglandin H2 (PGH2), a precursor of PGE2, while the latter is a terminal enzyme converting PGH2 to PGE2. PGE2 is relevant to photoaging research, as its downstream effects include inflammation, and to an extent, ECM remodeling in skin cells; both aspects are induced by external stressors such as UV.^53^, ^54^ While PTGES can be directly linked to the impact of UV on PGE2, as it is the related terminal enzyme, cyclooxygenases are less explored. In skin keratinocytes and fibroblasts, the mechanism of photoaging has previously been observed via ROS‐induced activation of cyclooxygenases; however, most studies have focused on the activation of cyclooxygenase‐2 (COX2) due to its better specificity for inflammation. It is generally regarded that COX1 is continuously produced regardless of external stressors as a homeostatic regulator.^55^ However, new insights through RNA‐seq indicate that COX1 may have a larger role in UV‐mediated inflammation.^25^

For prostanoid biosynthesis genes, PTGS1 was alleviated by 15–29% with formulations and PTGES was reduced by 56–74% (Figure 3). As the inorganic UV filters, partnered with the thickeners within the formulation, account for a thicker film of sunscreen, a lower dose of UV reaches the fibroblasts due to increased reflection, refraction, and absorption of light; less ROS is generated as a result. When UV penetrates the skin, NAPDH oxidase is increased and electrons are transferred from NAPDH to O_2_, thus generating ROS.^54^ In turn, ROS can stimulate the transcription of cyclooxygenases and downstream PGE2, as previously reported.^54^, ^56^ Increased PGE2 would trigger the inflammatory response and indirectly cause degradation of collagen.^53^, ^57^ By minimizing UV exposure which activates the *prostanoid biosynthesis* pathway upstream through ROS, PGE2 synthesis and stimulation of the inflammatory response would not be required. In other words, PTGS1 and PTGES alleviation would be greater.

In terms of protein expression, the significance of reduction in PTGES appears less pronounced based on immunocytochemistry and Western blotting (Figure 6). The immunocytochemistry images acquired showed that PTGES was expressed within the cytoplasm of most fibroblasts, regardless of protection with formulations. While the formulations appeared to cause a drop in the number of fibroblasts emitting higher intensity fluorescence in a single field of view in the images, broad localization and overlapping of cells rendered any attempt to quantify as biased. It was therefore difficult to ascertain the reduction caused by the formulations, based on the images alone. On the contrary, Western blot was able to quantify protein expression and identify that the formulations mostly alleviated PTGES; however, the extent of PTGES reduction was less than observed in gene expression. Regardless, it was possible to determine that the formulations did manage to minimize PTGES upregulation at both a gene and protein level to various extents. The lack of correlation between the magnitudes of reduction in gene and protein expression may be due to various factors such as translational regulation, protein degradation, posttranslational modifications, and experimental differences.^58^, ^59^

The three formulations alleviated PTGES to a greater extent in the complete solar condition compared with UV‐only at a genetic level. However, when investigating protein expression as a quantitative value through Western blotting, the same trend between irradiation conditions was only observed for F(TiO_2_^179nm^). F(ZnO^57nm^) and F(ZnO^57nm^/TiO_2_^47nm^) both showed an increased reduction in PTGES in the UV‐only condition by an additional 8% and 6% respectively; however, this difference is minimal in retrospect. It is understood in the literature that UV wavelengths can stimulate prostaglandin biosynthesis through UV‐induced ROS, which involves upregulation of PTGES to synthesize the inflammatory biomarker PGE2 from PGH2 in the cytoplasm.^56^, ^60^ The immunofluorescence images acquired in this study show that PTGES is broadly found in the cytoplasm and not localized to a specific area or organelle. The bulk of PTGES reduction, at a gene and protein level, in formulation‐protected fibroblasts would be caused by absorption, reflection, and refraction of UV wavelengths by TiO_2_ and ZnO particles. Direct links have not been established between visible light and PTGES.

As it is currently known that visible light wavelengths up to 700 nm can induce ROS generation, ROS‐mediated activation of the prostanoid biosynthesis pathway could occur with visible light.^61^ When UV and visible light are combined, ROS production is also understood to be amplified.^62^ Upon exposure to complete solar light, PTGES upregulation in unprotected fibroblasts is more pronounced than with UV‐only, which may be a result of amplified ROS. When fibroblasts are protected with the formulations, the resultant alleviation would be higher as the synergistic increase in ROS would be prevented when UV is blocked by TiO_2_ and ZnO.

### Formulations significantly alleviate irradiation‐induced changes in cell cycle genes by preventing p53/p21 signaling

The four cell cycle genes investigated, MDM2, CDKN1A, CCNE2, and SMAD3, all contribute to cell cycle regulation. MDM2 codes for mouse double minute 2 (MDM2) protein, which is a negative regulator of p53. CDKN1A codes for p21, which promotes cell cycle arrest. CCNE2 codes for cyclin E2, which stimulates cell cycle progression, as observed in multiple cell types.^63^, ^64^ Lastly, SMAD3 is involved with TGF‐β‐mediated transcription of GADD45, which promotes cell cycle arrest. All genes are involved in p53 signaling and cell cycle G1/S checkpoint regulation.^25^ In this study, the trends in upregulation and downregulation of the cell cycle genes suggest p53/p21‐mediated cell cycle arrest in response to UV.^11^ MDM2 and CDKN1A were upregulated in unprotected fibroblasts, as both genes promote cell cycle arrest, while CCNE2 was downregulated as it promotes cell cycle transition. While SMAD3 indirectly promotes cell cycle arrest through GADD45, its downregulation may be linked to the negative interactions of UV with TGF‐β, which mediates SMAD3.^65^

In the current study, photoprotection with inorganic filters caused an alleviation in MDM2 upregulation by 52–58%, CDKN1A upregulation by 69–77%, CCNE2 downregulation by 38–61%, and SMAD3 downregulation up to 20% (Figure 4). The mechanism of action for this alleviation would primarily be due to the action of TiO_2_ or ZnO particles directly absorbing, reflecting, or refracting UV light, which prevents direct or ROS‐mediated p53 and p21‐mediated downstream signaling.^66^, ^67^, ^68^ This can be directly associated with the alleviations of MDM2 and CDKN1A, as MDM2 is a negative regulator of the p53 protein and CDKN1A directly codes for the p21 protein.^66^ Both signaling pathways respond to UV by causing cell cycle arrest. In cell biology, p53 is the upstream response to external stressors, while p21 is a downstream response following the former.^69^ By blocking the dose of UV penetration with TiO_2_ and ZnO, the fibroblasts become less stressed, and these two signaling pathways do not over‐activate.

Contrary to the positive effects of TiO_2_ in sunscreens, multiple studies have found that TiO_2_ nanoparticles, if absorbed in the body, undesirably trigger genes regulated by p53, such as p21 and MDM2, in various cell types.^70^, ^71^, ^72^ Therefore, to circumvent this issue, it must be coated with alumina stearate in sunscreen application. The use of alumina stearate coating improves its biocompatibility, stability, and aesthetic appeal.^73^, ^74^ Furthermore, it is mandated by the European Commission^26^ In this study, all TiO_2_ particles are precoated with alumina stearate.

As irradiation‐induced p21 was the most impacted by the inorganic formulations, its protein expression was also observed (Figure 7). There was a high correlation between formulation‐protected CDKN1A (p21) gene and protein expression, with TiO_2_ and ZnO instigating >50% reduction. The formulations consistently demonstrated a greater impact against the UV‐only condition as UVB and UVA are the wavelengths that primarily induce p53 signaling; an upstream effector of p21. Both wavelength ranges can be blocked by TiO_2_ and ZnO which prevents direct DNA damage and ROS production. Downstream, p21 is a key regulator of the cell cycle. In the presence of DNA damage or ROS, cell cycle arrest is stimulated by activating p21 to prevent replication of damaged DNA and enable DNA repair.^69^, ^75^ In formulation‐protected fibroblasts, there would be less DNA damage and ROS production due to the sunscreen filters absorbing, reflecting, or refracting UVB and UVA; thus, p21 would not need to be upregulated.

CDKN1A can transcribe mRNA for p21 protein in the nucleus or the cytoplasm, as observed in the immunofluorescence images. With regard to photoaging and UV damage, it is nuclear p21 that binds to cell cycle‐promoting CDKs and causes cell cycle arrest.^76^ The key CDKs that are inhibited by p21 include CDK1, CDK2, and CDK4/6 complexes.^77^ Furthermore, nuclear p21 complexes with p53 and causes apoptosis, which can occur independently of cell cycle arrest but may become induced by it at G1/S and G2/M checkpoints.^78^ Contrary to the role of nuclear p21, cytoplasmic p21 is less studied. It has been reported to stimulate the opposite effects, mainly inhibiting apoptosis, promoting cell cycle progression, and promoting migration.^79^ In terms of UV induction and formulation protection, nuclear p21 expression is targeted rather than cytoplasmic p21 in dermal fibroblasts, as visualized by changes in nuclear fluorescence in immunocytochemistry (Figures 58A and 60A).

In the UV‐only condition, F(TiO_2_^179nm^) is most effective at reducing p21 protein expression, while in complete solar, it is F(ZnO^57nm^/TiO_2_^47nm^) that produces a greater reduction in p21. The first effect occurs as F(TiO_2_^179nm^) contains only TiO_2_, which has greater efficacy against UVB wavelengths rather than UVA.^15^ As p21 upregulation is more prominently caused by UVB, pure TiO_2_ formulations with a larger particle size to block UVB will likely incur better results. On the contrary, the second effect occurs because F(ZnO^57nm^/TiO_2_^47nm^) utilizes two different inorganic particles to potentially broaden the range of photoprotective action.^15^, ^17^ This benefit coincides with a wider range of wavelengths in the complete condition, as visible light and IR wavelengths are believed to synergize and amplify UV‐induced effects.^62^ Theoretically, F(ZnO^57nm^/TiO_2_^47nm^) would be able to strongly counter both UVB and UVA‐induced p21 upregulation due to greater photocatalytic ability across a broader range of wavelengths.

### Inorganic‐only (ZnO + TiO_2_ ) formulations provide slightly greater photoprotection at a gene expression level

F(ZnO^57nm^/TiO_2_^47nm^) is an inorganic‐only (ZnO + TiO_2_) formulation only achievable in formulations. It contained two types of inorganic UV filters at different active levels: 21.4%/2.4%, respectively (total active level of the formulation = 23.8%). The active levels for each of the inorganic UV filters total to under 25%, following European regulations.^26^ Multiple studies have previously considered or attempted to combine the action of TiO_2_ and ZnO into a single composite formulation, with the aim of broadening the UV target spectrum and enhancing UV absorption.^15^, ^17^, ^80^ Inorganic‐only (ZnO + TiO_2_) formulations, as a result, can benefit from greater UVA protection from ZnO and greater UVB protection from TiO_2_.^16^

Based on the gene expression profiles acquired, F(ZnO^57nm^/TiO_2_^47nm^) caused the greatest mean or maximum alleviation in irradiation‐induced MMP1, MMP3, PTGS1, PTGES, CDKN1A, and CCNE2, regardless of UV‐only or complete solar (Figures 2, 3, 4, 5); this constitutes the bulk of the genes identified. Therefore, the combined action of TiO_2_ and ZnO is evident at a genetic level. As observed in the absolute irradiance spectra for formulations (Figure 1), F(ZnO^57nm^/TiO_2_^47nm^) displays excellent UV protection from 290 nm to 374 nm, thus asserting that the induction of the above genes will primarily be caused by this wavelength range, defined as UVB and most of UVA. TiO_2_ and ZnO would ideally be a combination that provides broad UV protection up to 400 nm; however, its nanoparticle size would shift the protection spectrum slightly toward UVB.^15^, ^33^ Therefore, some UVA protection would inherently be lost.

## CONCLUSION

Formulations containing titanium dioxide and zinc oxide particles were found to alleviate the irradiation‐induced expression of eight photoaging‐associated genes which play a role in ECM modeling and degradation, prostaglandin‐mediated inflammation, and cell cycle checkpoint regulation. Significant alleviations were observed in the UV and complete solar light‐induced expression of seven genes, particularly the cell cycle genes MDM2, CDKN1A, and CCNE2. The photoprotective impact of the inorganic formulations also translated positively to the protein level, in the case of MMP1, PTGES, and p21. Although no statistical differences were observed in photoprotection between the formulations, the inorganic‐only (ZnO + TiO_2_) formulations exhibited the greatest mean or maximum alleviation in 75% of the genes investigated. The outcome of this study provides further insights on the photoprotective impact of inorganic particles in sunscreens, based on relevant signaling pathways, genes, and proteins that are induced by UV to accelerate photoaging.

## CONFLICT OF INTEREST STATEMENT

The authors declare no conflicts of interest.

## Data Availability

The data that support the findings of this study are available on request from the corresponding author. The data are not publicly available due to privacy or ethical restrictions.
